# Seizure control status and associated factors among pediatric epileptic patients at a neurologic outpatient clinic in Ethiopia

**DOI:** 10.1371/journal.pone.0259079

**Published:** 2021-11-03

**Authors:** Habtamu Digis Adal, Kassahun Alemu, Esileman Abdela Muche

**Affiliations:** 1 Department of Pharmacy, Teda Health Sciences College, Gondar, Ethiopia; 2 Department of Epidemiology and Biostatistics, Institute of Public Health, College of Medicine and Health Sciences, University of Gondar, Gondar, Ethiopia; 3 Department of Clinical Pharmacy, School of Pharmacy, College of Medicine and Health Sciences, University of Gondar, Gondar, Ethiopia; University of Rome Tor Vergata, ITALY

## Abstract

**Background:**

Childhood epilepsy is a major public health problem worldwide. Even though anti-seizure medications (ASM) have been demonstrated to control seizures, children with epilepsy continue to have frequent seizures. There is a scarcity of data on seizure control status among pediatric epileptic patients in Ethiopia. The aim of this study was to assess seizure control status and associated factors among pediatric epileptic patients.

**Methods:**

A hospital based cross-sectional study was done on 261 pediatric epileptic patients being followed up at the University of Gondar Comprehensive and Specialized Hospital, Northwest Ethiopia, from May 11 to August 11, 2019. The data were collected through caregiver interviews and patients’ medical records. All independent variables with p value of ≤ 0.2 in univariate analysis were taken to multivariable analysis. Adjusted odds ratio (AOR) with a 95% confidence interval was computed to see the predictors of seizure control status. Level of statistical significance was declared if *p*-value < 0.05.

**Result:**

From a total of 261 patients, 159 (60.9%) were males and had a mean age was 10.16 ± 4.62 years. More than half (57.1%) of participants were urban dwellers. Almost all (98.5%) were diagnosed with generalized tonic-clonic seizures. Majority (75.1%) of the patients were on mono-therapy. Forty-six (17.6%) of the patients reported adverse effects related to ASMs. Two hundred-three (77.8%) of the patients were adherent to their medications. Nearly half (49.0%) of the patients had controlled seizures. Urban residency (AOR: 2.12; 95% CI: 1.15–3.89; P = 0.016), adherence to medication (AOR: 3.92; 95% CI: 1.84–8.36; P < 0.001), use of combined ASM(AOR: 0.29; 95% CI: 0.14–0.59; P = 0. 001), durations of ASM use [2–5 years (AOR: 5.81; 95% CI: 2.89–11.70; P <0.001) and >5years (AOR: 4.80; 95% CI: 1.91–12.09; P = 0.001)]were significantly associated with seizure control status.

**Conclusion:**

Inadequate numbers of pediatric epileptic patients’ at the Ethiopian neurologic clinic achieved seizure control. Coming from a rural area, non-adherence to ASMs, use of multiple ASMs, and the use of ASMs for <2years were found to have a significant association with poorer seizure control, needing special attention to get control of seizure. Caregivers should closely monitor and address any barriers that contribute to ASM non-adherence and adverse drug events.

## Introduction

Epilepsy is the most common neurological disorder in children and a major public health problem worldwide. The seizures may start at any point during child’s lifetime and occur frequently [1]. The incidence of epilepsy is about 8 per 1,000 children below the age of 7 years. Around 5–10% of children suffer at least one seizure in the first 16 years of life. The incidence is highest in children below 3 years of age, with a decreasing frequency in older children. Studies reveal that globally nearly 150,000 children will sustain a first-time unprovoked seizure every year. From those, 30,000 will develop epilepsy [2]. The risk of premature death in children with epilepsy (CWE) is 5–10 times higher than in the general population. Global annual mortality rates range between 2.7 and 6.9 deaths per 1,000 CWE. In Africa, the childhood mortality rate is 92 per 1,000 live births; the distribution of deaths in CWE younger than 5years of age is 33% in south Asia, 50% in sub-Saharan Africa, and less than 1% in high-income countries [3–6]. Around 2 in 5 children and adolescents with seizures have a learning disability and 20%–30% of them had an intellectual disability, attention deficit hyperactivity disorder and other developmental disorder. Nearly two third (65.5%) of CWE discontinue their school [7, 8]. Furthermore poorly controlled seizure leads to impaired of quality of life, excessive bodily injury, impaired neuropsychological function, reduced marriage rates, poor education, reduced employment levels, reduced the capacity to participate in social activities, and shortened lifespan [9, 10]. Epilepsy in Ethiopia is also strongly associated with poor education and markers of poverty [11, 12].

Treatment with anti-seizure medication (ASM) is selected based on the type of seizure. The most recent recommendation is start ASMs either: after the second seizure or if the clinician believes that there is a >60% chance of the patient experiencing a second seizure, or if a diagnosis of an epileptic syndrome is made [13]. Treatment should be aimed at controlling seizures associated with the lowest possible overall cost and occurrence of adverse effects, thus allowing the child to become an active member of the community [14]. Although the number of ASMs increased globally and in Ethiopia, over 25% of CWE continue to have seizures [15]. ASMs fully control seizures in approximately 60% of children. The others 10–50% of children will gain good control of their seizures after a trial with second ASMs [16].

The seizure control capacity of ASM therapy in pediatrics patients depends on numerous factors, including, but not limited to, the availability and selections of ASMs, close monitoring of and the identification of underlying cause, the type of seizures, the pharmacokinetic parameters of ASMs, the caregiver’s knowledge, and attitude, and the level of adherence [8, 14].

Most of the previous studies in Ethiopia were carried out on adults with epilepsy, while the present study focused on CWE aged 1–18 years. Identifying factors that contribute to the seizure control status of CWE is very important and requires a deeper understanding. This helps to develop new approaches to improve the utilization of health care services and seizure control. This study therefore aimed to describe the treatment outcomes of childhood epilepsy at University of Gondar Comprehensive and Specialized hospital (UoGCSH).

## Methods

### Study area

The study was conducted at the pediatric neurologic chronic outpatient department (OPD) clinic of UoGCSH, Northwest Ethiopia. The hospital is located in the Amhara National Regional State, 735 km away from Addis Ababa, the capital city of Ethiopia. It is one of the biggest tertiary-level teaching and referral hospitals in the region. The hospital serves more than seven million people in North Gondar and neighboring administrative zones. The catchment area comprises of rural and urban areas. The pediatric neurology clinic is an outpatient clinic that treats patients ≤18 years old. The clinic provides once weekly service (i.e. every Friday) to a total of 308 pediatrics epileptic patients on follow up basis. The health care providers at the paediatrics neurologic clinic includes one pediatric neurologist, 2–3 neurology residents, 3–4 intern physicians, and 2 nurses. The available resources for the diagnosis and treatment of epilepsy includes computed tomography (CT) scan, magnetic resonance imaging (MRI), electro encephalogram (EEG), random blood sugar and electrolyte tests.

### Study design and period

The study was a hospital-based cross-sectional study conducted from May 11 to August 11, 2019.

### Population

#### Source population

- All pediatric epileptic patients attending the chronic follow-up clinic of UoGCSH

#### Study population

- All pediatric epileptic patients attending the chronic follow-up clinic of UoGCSH and fulfilling the inclusion criteria during the study period.

### Inclusion and exclusion criteria

#### Inclusion criteria

- Patients of age ≤ 18 years and diagnosed to have epilepsy,
- Patients who were on ASMs for at least a year, and
- Patients whose caregivers were willing to give informed consent.

#### Exclusion criteria

- Pediatric patients having seizures induced by drugs or due to any trauma or disease,
- Febrile seizure, and
- Unreadable and incomplete medical records were excluded from the study.

### Sample size and sampling techniques

The total numbers of pediatrics epileptic patients on follow-up at the chronic outpatient clinic of UoGCSH were small (308) so; all eligible patients were included in this study. A total of 261 pediatrics epilepsy patients were included in the current study [17].

### Data collection tool

Data was collected by using a structured questionnaire which contains three parts (S1 Text). Part I was about participant’s demography, while Part II contained questions regarding medication adherence and barriers for non-adherence. Part III contained questions about patient’s medical history and medication information. The questionnaire was first prepared in English and translated to Amharic (except part III), the local language, and then retranslated to English to ensure the consistency of the tool.

Medical and medication information were retrieved by reviewing each patient’s medical records using data extraction form (part III). The data extraction form contains the following information: the date of registration (hospital visit date), the date of ASMs initiation use, the patients’ age at the diagnosis of epilepsy, diagnosis or seizure type, patients follow up time, number of seizure episodes per month before ASMs used, seizure in the current follow up time, durations of seizure-free period, hospitalization or emergency care admission associated with epilepsy including seizure type/s, current ASM(s), number of ASMs, durations of ASMs used, any diagnosis other than epilepsy, any prescribed drugs other than ASM (s), and any adverse effect due to ASMs. The clinical information of the patients during the last one year also assessed.

Ten days before data collection, a pre-test was conducted on 15 pediatric epileptic patients on follow up at UoGCSH to ensure the completeness and consistency of the data collection tool. But those patients who participated in the pre-test were excluded from the main study.

#### Recruiting and training data collectors

Data were collected by two nurses and one general practitioner. The data collectors were trained about the objective of the study, and the methods of data collection including data extraction from patient charts as well as techniques of interviewing patients. The principal investigator (HDA) supervised the data collection throughout the process.

### Data processing and analysis

The collected data were entered in to Epi-Info^™^ 7 software and cleaned, checked for its completeness, categorized, and coded. Then it was transferred to Statistical Package for the Social Science (SPSS Version 21.0) and was analyzed. Descriptive statistics were done for demographic and clinical details. The results were presented in the form of tables and graphs. Both bivariable and multivariable binary logistic regression analyses were done to see factors associated with seizure control status. All independent variables with *p* value ≤ 0.2 in univariate analyses were taken to multivariable analysis to control for all the possible confounders.

Adjusted odds ratio (AOR) with a 95% confidence interval (CI) was computed to see the magnitude of association between seizure control status and the independent variables (gender, age, residence, education, adherence to ASMs, number of seizures before initiation ASMs, and the type and duration of ASM treatment). Level of statistical significance was set at *p*–value of < 0.05.

### Ethical considerations

Ethical clearance was obtained from ethical review committee of the school of pharmacy of University of Gondar. Letter of cooperation was obtained from UoGCSH clinical directorate before going to neurology clinic. Moreover, written informed consent was obtained from each primary care giver of patients involved in the study and verbal assent were taken from the CWE. Furthermore, privacy and confidentiality of the information about the patients were ensured during primary care givers’ interviews and review of patients’ chart. The data was used for the study purpose only. And no personal identifying information was recorded in the data extraction formats.

### Operational definition

Epilepsy was diagnosed clinically with history of two or more seizures that started in the brain with support of abnormal EEG finding [18]. In this study, the diagnosis of epilepsy is taken from each patient’s medical chart. Similarly, types of seizures were taken from the medical chart based on the EEG findings.

The seizure status was considered controlled if the patient had not experienced any seizure attacks in the past year, and not controlled if the patient experienced one or more seizure attacks in the last one year follow up period [19, 20].

Adherence was measured with pill count method where caregivers were asked to complete a questionnaire that helps to calculate the number of ASMs doses that were taken between appointments and comparing it with the total number of ASMS doses that the patient had received. = [(# of doses prescribed per follow up—# of doses missed per follow up)/# of doses prescribed per follow up)], which ranged from 0–100%. The patient was considered to be non-adherent if < 80% of prescribed pills were taken. On the other hand the patient who took > 80% of prescribed pill were considered to be adherent [21].

## Results

### Socio-demographic characteristics

**Fig 1:**
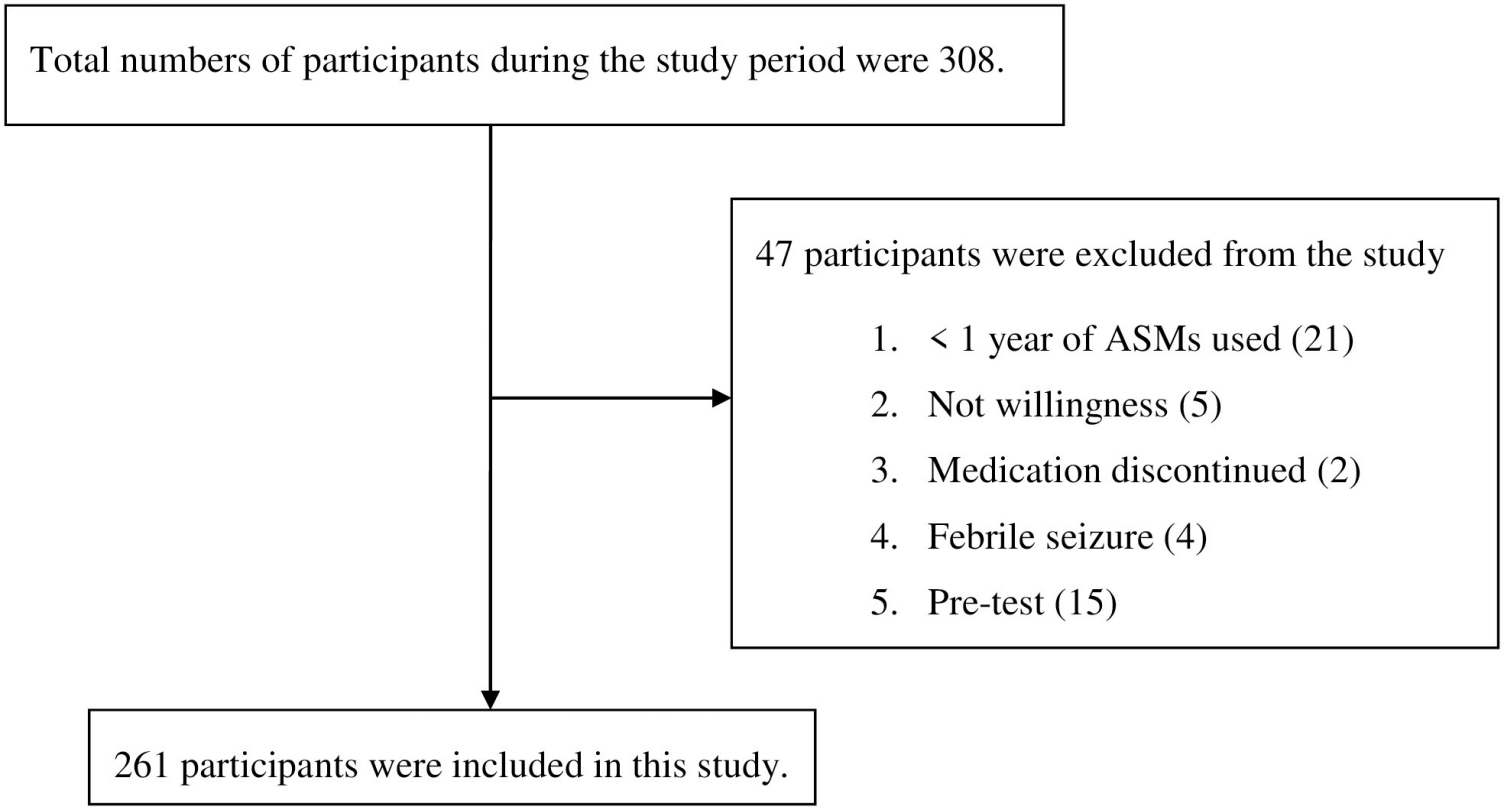
Participant selection criteria of pediatric epileptic patients on follow-up at UoGCSH, Northwest Ethiopia, from May 11 to August 11, 2019 (N = 261). ASM, Anti seizure medications.

A total of 261 patients were included in this study (Fig 1). The mean age of participants was 10.15 ± 4.6 years. The majority of the patients were males (60.9%), urban dwellers (57.1%), and Orthodox Christians (89.7%). Ninety percent of patients had no family history of epilepsy. More than half of the study participants (52.5%) were attending primary school at the time of data collection (Table 1).

**Table 1:** Socio-demographic characteristics of pediatric epileptic patients on follow-up at UoGCSH, Northwest Ethiopia, from May 11 to August 11, 2019 (N = 261).

Variables	Category	Frequency n, (%)
Gender	Female	102 (39.2%)
	Male	159 (60.9%)
Age	1–5	58 (22.3%)
	5.1–10	64 (24.5%)
	10.1–15	105 (40.2%)
	15.1–18	34 (13.0%)
Residency	Urban	149 (57.1%)
	Rural	112 (42.9%)
Religion	Orthodox Christian	234 (89.7%)
	Islam	27 (10.3%)
Family history	No	235 (90.0%)
	Yes	26 (10.0%)
Educational level	Pre-school	114 (43.7%)
	Primary school	137 (52.5%)
	Secondary school	10 (3.8%)
Health care payment method	Self	158 (60.5%)
	Health insurance	103 (39.5%)

### Clinical characteristics

Among the study participants, nearly half (49.0%) of patients had controlled seizure. GTCS was the most common (98.5%) type of seizure. About three-fourths (75.1%) of the patients were on mono-therapy. Nearly half (47.5%) of participants had more than eight episodes of seizure per month before initiation of ASMs Forty-six (17.6%) patients reported that they had experienced at least one adverse effect related to their ASMs therapy and 8 patients reported that they had more than one adverse drug effect: (weakness & irritability(n = 3), depressed mood & irritability(n = 2), headache & irritability(n = 1), depressed mood & headache (n = 1), epigastric pain & nightmare(n = 1)). Two hundred-three (77.8%) patients were adherent to their medications. The clinical characteristics of participants are summarized in Table 2.

**Table 2:** Clinical characteristics of pediatric epileptic patients on follow-up at UoGCSH, Northwest Ethiopia, from May 11 to August 11, 2019 (N = 261).

Variables	Category	Frequency n, (%)
Age at diagnosis of epilepsy (years)	0–5	101(38.7)
	5.1–10	107 (41.0)
	10.1–17	53 (20.3)
Types of seizure	GTCS	257 (98.5)
	Atonic Seizure	2 (0.8)
	Unclassified seizure	2 (0.8)
No of seizure episodes per month before ASMs used	1–2	76 (29.1)
	3–5	49 (18.8)
	6–8	12 (4.6)
	>8	124 (47.5)
Number of ASM(s) used	Mono-therapy	196 (75.1)
	Combination therapy	65 (24.9)
Type of ASM(s) used	PHB	107 (41.0)
	PHB+PHT	38 (14.6)
	PHB+VPA	18 (6.9)
	PHT	71 (27.2)
	PHT+VPA	9 (3.4)
	VPA	18 (6.9)
Durations of ASMs used (years)	1–2	89 (34.1)
	2.1–5	117 (44.8)
	>5	55 (21.1)
Follow up time (appointment)	Every One Month	172 (65.9)
	Every Two Months	73 (28.0)
	Every Three Months	16 (6.1)
Seizure in the current follow up time	No	186 (71.3)
	Yes	75 (28.7)
Seizure free years	<1	133 (51.0)
	1–2.0	105 (40.2)
	2.1–5	23 (8.8)
Seizure control status	Uncontrolled	133 (51.0)
	Controlled	128 (49.0)
Diagnosis other than epilepsy	No	258 (98.9)
	Yes	3 (1.1)
Drugs prescribed other than ASMs	No	259 (99.2)
	Yes	2 (0.8)
Adverse effects	No	215 (82.4)
	Yes	46 (17.6)
Type of adverse effect (n = 54)	Irritability	16 (29.6)
	Forgetfulness	8 (14.8)
	Gingival hyperplasia	8 (14.8)
	Headache	6 (11.1)
	Depressed mood	6 (11.1)
	Confusion	3 (5.6)
	Weakness	3 (5.6)
	Epigastric pain	2 (3.7)
	Nightmare	1 (1.9)
	Skin rash	1 (1.9)
Adherence status	Non-adherent	58 (22.2)
	Adherent	203 (77.8)

GTCS, Generalized tonic clonic seizure; PHB, Phenobarbitone; PHT, Phenytoin; VPA, Valpuroic acid

A total of 58 (22.2%) patients were non adherent to their ASMs. The top three barriers for adherence were the child’s refusal to take the medicine 17(6.5%), lack of benefit 16(6.1), and forgetfulness to take medication 15(5.7%). The results are summarized in Table 3.

**Table 3:** Reasons for non adherence among pediatric epileptic patients on follow-up at UoGCSH, Northwest Ethiopia, from May 11 to August 11, 2019 (N = 93).

Barriers for adherence	Number of patient n (%)
The child refuses to take the medicine	17 (18.3%)
Lack of benefit	16 (17.2%)
Forget to take medication	15 (16.1%)
Improvement and seizure free periods	14 (15.1%)
High frequency of medication	14 (15.1%)
Side effects of the medications	7 (7.5%)
Poor medication counseling	3 (3.2%)
Embarrassed to take medicine in front of friends or family	3 (3.2%)
The pill is difficult for administration or difficult to swallow	2 (2.2%)
Child does not like the taste of medicine.	1 (1.1%)
High cost of medications	1 (1.1%)
Total	93 (100.0%)

### Factors associated with seizure control status

Bivariable logistic regression analysis did not show any association between seizure control and family history of epilepsy, health care payment, age at the diagnosis of epilepsy, follow up appointment period and adverse effect of ASMs. After adjusting for the above mentioned variables the final multivariable binary logistic regression analyses showed that urban residency (AOR: 2.12; 95% CI: 1.15–3.89), adherence (AOR: 3.92; 95% CI: 1.84–8.36), combined ASMs use (AOR: 0.29; 95% CI: 0.14–0.59), ASMs use for 2–5 years (AOR: 5.81; 95% CI: 2.89–11.70), and ASMs use for more than 5 years (AOR: 4.80; 95% CI: 1.91–12.09) were associated with seizure control (Table 4).

**Table 4:** Logistic regression analysis result of factors associated with seizure control status among pediatric epileptic patients at UoGCSH, Northwest Ethiopia, from May 11 to August 11, 2019 (N = 261).

Variable	Category	Treatment outcome		COR (95% CI)	AOR(95%CI)	*p*-value
		Uncontrolled seizure n (%)	Controlled seizure n (%)			
*Gender	Female	45 (44.1%)	57(55.9%)	1	1	1
	Male	88 (55.3%)	71(44.7%)	0.64 (0.39–1.05)	0.66 (0.36–1.19)	0.168
*Age (years)	1–5.0	39 (67.2%)	19 (32.8%)	1	1	1
	5.1–10.0	32 (50.0%)	32 (50.0%)	2.05 (0.98–4.28)	1.66 (0.61–4.50)	0.322
	10.1–15.0	43 (41.0%)	62 (59.0%)	2.96 (1.51–5.80)	1.97 (0.69–5.60)	0.206
	15.1–18	19 (55.9%)	15 (44.1%)	1.62 (0.68–3.87)	0.82 (0.20–3.39)	0.787
*Residence	Urban	68 (45.6%)	81 (54.4%)	1.65 (1.00–2.70)	2.12 (1.15–3.89)	0.016*
	Rural	65 (58.0%)	47 (42.0%)	1	1	1
*Educational level	Pre-school	88(55.3%)	71(44.7%)	1	1	1
	Primary School	45 (44.1%)	57(55.9%)	2.58 (1.54–4.30)	1.44 (0.65–3.17)	0.366
	Secondary School	4(40%)	6(60%)	2.67 (0.71–10.02)	1.86(0.35–10.01)	0.469
* Adherence	Non-adherence	42 (72.4%)	16 (27.6%)	1	1	1
	Adherence	91(44.8%)	112 (55.2%)	3.23 (1.71–6.12)	3.92 (1.84–8.31)	<0.001*
*No of seizure episodes per month before ASMs used	1–2	28 (36.8%)	48 (63.2%)	1	1	1
	3–5	24 (49.0%)	25 (51.0%)	0.61 (0.29–1.26)	0.53 (0.22–1.28)	0.159
	6–8	7 (58.3%)	5 (41.7%)	0.42 (0.12–1.44)	0.37 (0.09–1.54)	0.173
	>8	74 (59.7%)	50 (40.3%)	0.39 (0.22–0.71)	0.53 (0.26–1.09)	0.083
*Number of ASMs used	Mono-therapy	84 (42.9%)	112 (57.1%)	1	1	1
	Combination therapy	49 (75.4%)	16 (24.6%)	0.25 (0.13–0.46)	0.29 (0.14–0.59)	0.001*
*Durations of ASMs used (years)	1–2	63 (70.8%)	26 (29.2%)	1	1	1
	2–5	44 (37.6%)	73 (62.4%)	4.02 (2.23–7.26)	5.81 (2.89–11.70)	<0.001*
	>5	26 (47.3%)	29 (52.7%)	2.70 (1.34–5.44)	4.80(1.91–12.09)	0.001*

ASMs: Anti seizure medications, COR: Crude odds ratio AOR: Adjusted odds ratio, CI: Confidence interval

* Statistical significant association

## Discussion

In the current study, the overall treatment outcome revealed that nearly half (49.0%) of the participants had controlled seizures. This is lower than a study conducted in Ethiopia (77%) [22] Nigeria (64%) [20] and Norway (59.0%) [23]. The possible justification for this could be that the definition of controlled seizure in the other Ethiopian study was so broad that the result may be inflated (3 or fewer seizure during three month period). On the other hand differences in the time of initiating ASMs treatment, selections of ASMs, seizure type, cause of epilepsy, pharmaco-genomic factors, drug resistance and other associated factors that lead to poor response to treatment may contribute to the higher rates of controlled seizure in the Norwegian study [24].

Epileptic seizures were found to be more common in males (60.9%) than in females (39.1%). This is in line with a study done in Kenya [25]. Less than half of the patients (40.2%) belonged to the age group 10 to 15 years. This is different from the other studies carried out in Malaysia, Kenya and India in which a majority of children fall in the age group of 6 to 10 year [14, 25, 26].

The choice of the most suitable ASMs depends on the correct diagnosis of the seizure type. GTCS was the most common type of epileptic seizure encountered in 98.5% of patients. This was higher than that was reported by studies done in India (55.0% and 55.2%), Malaysia (40.0%), and Ethiopia (48.6%) [14, 26–28]. The possible justification for this could be due to the difference in qualification of expertise and the diagnostic tools used, which can facilitate the classification of seizure type [29]. Also, low level of public awareness towards partial seizure, poor access to diagnostic imaging which helps health care providers to differentiate the seizure types, and lack of qualified experts in the study setting may all contribute to the observed differences with the other studies.

Multivariable binary logistic regression analysis revealed factors associated with seizure control status. Patients from urban area were 2.12 times more likely to have controlled seizure compared to patients from rural residence. Of those patients who live in urban areas, 54.4% of them had controlled seizure this could be explained by urban residents’ better awareness about the disease which facilitates a discussion with their health care professionals about the disease and the medications they use. In addition, most rural residents could be inclined to try religious and spiritual healing methods, reflecting the traditional thinking and beliefs about the disease [30, 31]. This condition is also bad in rural and remote areas where almost no services for epilepsy are accessible [5, 32].

Adherent patients were almost 4 times more likely to have controlled seizure than non-adherent patients. In agreement to this study, many studies have shown that adherence to medications improved the effectiveness of interventions [33]. Patients face different barriers that may contribute to their non-adherence. For this study, the top three barriers for adherence were the child’s refusal to take the medicine 17(6.5%), lack of benefit 16(6.1), and forgetfulness to take medication 15(5.7%). This finding is in line with the study conducted in Jordan [34], where the top 3 reasons for non-adherence were forgetfulness, children refusal to take medication and dissatisfaction with their medication.

Patients who used ASMs for 2 to 5 years duration were nearly 6 times more likely to have controlled seizure as compared to patients who took ASMs for 1 to 2 years. Patients who took ASMs for more than 5 years had 4.80 times more control seizure when compared with those patients who took ASMs for 1 to 2 years. Of those patients who used ASMs more than 5 years duration, 52.7% had controlled seizures and only 29.2% of patients who used ASMs for 1 to 2 years had controlled seizures. This shows that a longer duration of ASMs use associated with the seizure control status [35].

Patients who use combination therapy for their epilepsy had 71.0% less likely to have controlled seizure when compared with patients who were on mono-therapy. Of those patients who were on mono-therapy, 57.1% had controlled seizure and only 24.6% of combination therapy users had controlled seizures. In this finding, patients taking combination agents were less likely to control their seizure status. The lower level of adherence as a result of the prescription of multiple medications could contribute to the lower seizure control in patients on multiple ASMs when compared to those on monotherapy. In support of this justification adherence was shown to positively associate with seizure control status in this study. And most importantly, a patient with a more intractable epilepsy syndrome would be taking multiple ASMs [36].

Antiepileptic drug regimen is defined as a trial of either a single drug (mono-therapy) or a combination of two or more of ASMs treatment. Majority of patients with epilepsy respond to one of the first-line ASMs. Second-line drugs may be useful in patients who do not respond to one or a combination of the first- line ASMs [37, 38]. Mono-therapy was found to be the therapy of choice for majority of pediatric epileptic patients (75.1%) due to the fact that milder epilepsy only requires one agent. The findings of this study is in line with 2 studies conducted in India (71.0% and 73.5%), and less than what was reported in another study conducted in Ethiopia (88%) [26–28]. However, this is higher than study carried out in Malaysia (54.3%) [14]. The justification behind this could be, in Malaysia, health care professionals tend to prescribe more combination ASMs (45.7%) than what is reported in our setting (24.9%).

In the present study, phenobarbitone (41.0%) was found to be the most frequently prescribed drug as mono-therapy. The finding of the present study is different from studies done in different places, where carbamazepine and valproate were the most commonly prescribed ASMs in India [26, 39] and Malaysia [14], respectively. This could be due to long term use history, safety profile, availability, and affordability of phenobarbitone. WHO also recommends phenobarbitone as the first line drug for almost all types of seizure in developing countries including Ethiopia [1, 5]. Differences in country specific treatment guidelines could be the reason for differences in prescribing practices across different countries.

In this study, 17.6% of patients reported that they had experienced adverse effects related to their ASMs therapy. This result is lower than the finding from a study carried out in Singapore in which the adverse effects of ASMs were reported by 76.3% of participants [32]. The reasons behind this could be lower rate of pediatric ADRs reporting and inadequate public health programs for medicine safety monitoring in Ethiopia [40].

### Limitation of the study

This study was done at single neurologic clinic North West part of Ethiopia with limited number of participants so it has potential limitation of generalizability of the work to Ethiopia as a whole. Also, the inclusion criteria state that patients must have been on ASMs for at least one year this is another potential limitation as the study population does not completely represent all people with epilepsy.

## Conclusion

Nearly half of pediatrics epilepsy patients’ attending an Ethiopian neurologic clinic had controlled seizure. Patients who came from rural area, were non adherent to their ASMs use combination of ASMs, and were on ASMs for less than two years were found to have significant association with seizure control and need special attention to get their seizure controlled. The caregivers have crucial roles to play in monitoring and addressing the possible barriers for non-adherence and adverse drug events.

## Supporting information

S1 Text Data abstraction form. (DOCX)

S1 Table Reported side effect of ASMs among pediatric epileptic patients on follow-up at UoGCSH, Northwest Ethiopia, from May 11 to August 11, 2019 (N = 46). (DOCX)

## Abbreviations

ASMs: Antiepileptic Drugs
CWE: Children with epilepsy
GTCS: Generalized Tonic-Clonic Seizure
OPD: Out Patient Department
SPSS: Statistical Package for the Social Science
UoGCSH: University of Gondar Comprehensive and Specialized Hospital
WHO: World Health Organization
