# Supplementary material for: Seizure control status and associated factors among pediatric epileptic patients at a neurologic outpatient clinic in Ethiopia
Source: PLoS One. 2021 Nov 3;16(11):e0259079. doi: 10.1371/journal.pone.0259079 (PMC8565750; doi:10.1371/journal.pone.0259079)
Supplement: S1 Text — (DOCX) [file pone.0259079.s001.docx]

**Supporting information**

**S1 Text: Data Abstraction Form**

**Part I: Patient’s demography**

1. Gender € Male € Female
2. Age = -----------Year/s
3. Residence € Urban € Rural
4. Religion: € Orthodox € Islam € Protestant € Other/s------------------
5. Dose the patient have Epileptic case of Family history? € Yes € No
6. Patient’s educational level: € pre-school € KG ----------- € Unable to read/ write € Primary School (grade 1 to 8) € Secondary School(grade 9 to 12) € Diploma

**Part II: Assessment of adherence**

**1.** Total number of prescribed pills: __________

2. Prescribed pill per day: ______________

3. Days since prescription filled date: _______________

4. Number of pills not taken:__________________

5. Percentage of adherence: _____________

6. Level of adherence: 0. poor 1. good

**Why the patient didn’t take the pills? (Barriers for adherence)**

| 1. Side effects of the medications. | 1. Poor medication counseling. |
| --- | --- |
| 1. Child does not like the taste of medicine. | 1. Improvement and seizure free periods. |
| 1. High cost of medications. | 1. Embarrassed to take medicine in front of friends or family. |
| 1. The pill is difficult for administration or difficult to swallow. | 1. High frequency of medication |
| 1. Forget to take medication. | 1. Other things, like sport or school, get in the way of taking my medicine. |
| 1. Lack of benefit. | 1. The child refuses to take the medicine. |
| 1. Others (if any): | |

**PART III: Data abstraction format from patient and medical records**

1. Date of registration (Hospital Visit): ----------------------------------------------------------
2. When did he/she start antiepileptic drug(s) treatment? ----------------------------------------
3. How old was the patient when he/she diagnosis as epilepsy? --------------
4. Diagnosis (seizure type) --------------------------------------------------------------------------.
5. What is the patient’s follow up time? Every ------------------ days/weeks/months
6. Number of seizure episode per month before antiepileptic drug therapy -------episode/s
7. Was there any seizure in the current follow up time? € Yes € No
8. If Q7 is YES, how many times? ------------------------------episode/s
9. If Q7 is **NO**; when did it stop? Before ----------------month/s or before---------------year/s
10. Was there any hospitalization or emergency care admission associated with epilepsy? € Yes € No,
11. If Q10 is YES: what was the case condition? Seizure type/s ----------------------------
12. Current antiepileptic drug(s) prescribed with their strength/s or dose (mg) and frequency (Daily, BID, TID ---).

| Drug name | Strength/s (Dose) | Frequency (Daily, BID, TID--------) |
| --- | --- | --- |
|  |  |  |
|  |  |  |
|  |  |  |

1. Durations of Medication/s was/were taken = ------------------ Year/s
2. Diagnosis other than epilepsy ----------------------------------------------------------------------
3. Drugs prescribed other than antiepileptic drug (s) -----------------------------------------------
4. Was there any experienced adverse effect of the AEDs? € Yes € No
5. If Q18 is **yes**, what type of adverse effect/s occurred?

| 1. Depressed mood | 1. Blurring of vision | 1. Skin rash |
| --- | --- | --- |
| 1. Epigastric pain | 1. Headache | 1. Irritability |
| 1. Confusion | 1. Nightmare | 1. Gingival Hyperplasia |
| 1. Weakness | 1. Forgetfulness | 1. Other/s--------------------- |

1. Year based follows up for Number of seizures:

| 18. 1. From May 23, 2018 to August 23, 2019. | | | | | | | | | | | | |
| --- | --- | --- | --- | --- | --- | --- | --- | --- | --- | --- | --- | --- |
| Months | Sept | Octo | Nove | Dece | Jan | Feb | Mar | Apr | Ma | Jun | Jul | Aug |
| No of seizures |  |  |  |  |  |  |  |  |  |  |  |  |
| Reason for number of seizure increasing (if any increased): | | | | | | | | | | | | |

| 18.2. Year: From May 23, 2017 to 2018. | | | | | | | | | | | | |
| --- | --- | --- | --- | --- | --- | --- | --- | --- | --- | --- | --- | --- |
| Months | Sept | Octo | Nove | Dece | Jan | Feb | Mar | Apr | Ma | Jun | Jul | Aug |
| No of seizures |  |  |  |  |  |  |  |  |  |  |  |  |
| Reason for number of seizure increasing (if any increased): | | | | | | | | | | | | |
