# Supplementary material for: Seizure control status and associated factors among pediatric epileptic patients at a neurologic outpatient clinic in Ethiopia
Source: PLoS One. 2021 Nov 3;16(11):e0259079. doi: 10.1371/journal.pone.0259079 (PMC8565750; doi:10.1371/journal.pone.0259079)
Supplement: S1 Table — (DOCX) [file pone.0259079.s002.docx]

**S1: Table**

Table: - Reported side effect of ASMs among pediatric epileptic patients on follow-up at UoGCSH, Northwest Ethiopia, from May 11 to August 11, 2019 (N = 46).

| Type Of ASMs | Reported Side Effect | No Patients Reported |
| --- | --- | --- |
| Phenytoin | Headache | 2 |
|  | Gingival Hyperplasia | 5 |
|  | Depressed Mood | 1 |
|  | Irritability | 2 |
| Phenobarbitone | Confusion | 3 |
|  | Irritability | 6 |
|  | Forgetfulness | 4 |
|  | Depressed Mood & Irritability | 1 |
|  | Weakness & Irritability | 2 |
|  | Depressed Mood | 1 |
|  | Gingival Hyperplasia | 1 |
|  | Headache | 1 |
|  | Headache & Irritability | 1 |
|  | Skin Rash | 1 |
|  | Depressed Mood & Headache | 1 |
| Valpuroic Acid | Headache | 1 |
|  | Depressed Mood & Irritability | 1 |
|  | Weakness & Irritability | 1 |
|  | Depressed Mood | 1 |
| Phenobarbitone + Phenytoin | Forgetfulness | 4 |
|  | Irritability | 1 |
| Phenobarbitone + Valpuroic Acid | Epigastric Pain & Nightmare | 1 |
|  | Epigastric Pain | 1 |
|  | Irritability | 1 |
| Phenytoin + Valpuroic Acid | Gingival Hyperplasia | 2 |

ASM: Anti seizure medications, +: And
